# Prevalence and intensity of soil-transmitted helminth infections in Uganda: Results from population-based prevalence surveys in five districts

**DOI:** 10.1371/journal.pntd.0011605

**Published:** 2023-09-26

**Authors:** Benjamin Tinkitina, Prudence Beinamaryo, Moses Adriko, Betty Nabatte, Moses Arinaitwe, Alfred Mubangizi, Paul Emerson, Sanjaya Dhakal, Kristin M. Sullivan

**Affiliations:** 1 Vector Borne and Neglected Tropical Diseases Division, Ministry of Health, Kampala, Uganda; 2 Children Without Worms, The Task Force for Global Health, Decatur, Georgia, United States of America; Federal University of Agriculture Abeokuta, NIGERIA

## Abstract

**Background:**

Soil-transmitted helminth (STH) infections are caused by roundworms (*Ascaris lumbricoides*), whipworms (*Trichuris trichiura*), and hookworms (*Necator americanus* and *Ancylostoma duodenale*). In Uganda, baseline surveys conducted during the late 1990s and early 2000s suggested STH infections were common, with prevalence >50% among surveyed schoolchildren. In 2003, a national program was launched with mass preventative chemotherapy (PC) and health education for children 1–14 years old. Little evidence is available to show the impact of national deworming.

**Methods:**

We conducted population-based, cross-sectional household surveys in five districts (Buikwe, Kassanda, Kiryandongo, Kisoro, and Rubanda) in March and May 2022. Our primary objective was to estimate STH prevalence by species due to infections of any intensity and infections of moderate-to-heavy intensity among preschool-aged children (PSAC, 1–4 years old), school-aged children (SAC, 5–14 years old), and women of reproductive age (WRA, 15–49 years old). Laboratory technicians used duplicate Kato-Katz microscopy to determine fecal egg count.

**Results:**

Overall, 3,352 PSAC; 3,884 SAC; and 1,226 WRA provided stool samples. The prevalence of any infection remained high in Kisoro at or above ~50% within all risk groups. In other districts, the prevalence of any infection ranged from approximately 5 to 16% among PSAC, 6 to 23% among SAC, and 12 to 19% among WRA. Moderate-to-heavy intensity infection prevalence was highest in Kisoro (~15–26%), followed by Rubanda (<5%), and was ≤1% in other districts. *A*. *lumbricoides* and *T*. *trichiura* infections were largely confined to Kisoro and Rubanda, whereas hookworm was most common in other districts.

**Conclusions:**

The STH prevalence has decreased markedly in three districts in Uganda. Based on our findings, the national deworming program should consider decreasing PC distribution frequency in these districts per the World Health Organization guidelines. Efforts are needed to understand why the Kisoro and Rubanda districts did not demonstrate similar gains.

## Author summary

Intestinal worm infections—formally known as soil-transmitted helminth (STH) infections—are a public health problem that can potentially reduce physical growth, cause anemia, and hinder cognitive development. In Uganda, baseline surveys conducted between 1998 and 2005 indicated that STH infection prevalence was over 50% among school-aged children in most surveyed districts. As a result of these findings, in the early 2000s, the Ugandan Ministry of Health began twice-a-year national deworming campaigns and health education among preschool- and school-aged children.

In March and May of 2022, we conducted follow-up surveys in five districts to determine how well the deworming campaigns have reduced infection prevalence in these communities. Among surveyed groups (preschool-aged children, school-aged children, and women of reproductive age), we found that in three of the five surveyed districts, STH prevalence had substantially decreased. In these areas, the national deworming program may consider reducing the frequency of the deworming campaigns from its current twice-a-year schedule. The prevalence remained high in the remaining two districts despite several years of deworming. Further investigation is needed to determine why.

## Introduction

Soil-transmitted helminth (STH) infections are neglected tropical diseases that pose a great and often silent burden of morbidity on poor populations in developing countries [1,2]. Most common helminth infections are caused by roundworms (*Ascaris lumbricoides*), whipworms (*Trichuris trichiura*), and hookworms (*Necator americanus* and *Ancylostoma duodenale*). More than 1.5 billion infections are widely distributed in tropical and subtropical areas, with the greatest numbers occurring in sub-Saharan Africa, the Americas, China, and East Asia [1]. Increased hand washing, improved sanitation, and access to safe water are thought to prevent transmission [3–6], although the evidence is mixed.

In Uganda, national baseline pre-treatment surveys conducted from 1998 to 2005 in 46 of 56 districts revealed that STH infections were widespread throughout the country, with the prevalence of any STH infection >50% among surveyed schoolchildren in >75% (35/46) of districts [7]. Similar to what was found in a prior analysis [8], marked heterogeneity in prevalence and dominant species was observed. The prevalence of *A*. *lumbricoides* and *T*. *trichiura* was highest in the southwest (with district-level prevalence generally above 80%), moderate in selected central and eastern districts, and there was a near absence of transmission in the arid northeastern districts (i.e., the Karamoja region). By contrast, hookworm was homogeneously distributed throughout the country, with prevalence in some areas exceeding 85% of surveyed sites, again with low prevalence in Karamoja.

With the majority of surveyed districts having a prevalence of any STH infection >50% among school-aged children, the entire country was targeted for twice-a-year preventative chemotherapy (PC) treatment according to the World Health Organization (WHO) guidelines [9,10]. A national control program was initiated in 2003 and began distributing PC to some parts of the country co-endemic with schistosomiasis (bilharzia) using single-dose albendazole 400mg or mebendazole 500mg. The control program was scaled-up to cover the entire country in 2005 through integrated “Child Health” days, where children between the ages of 1 and 14 years old were treated in schools and outreach posts. Twice-a-year deworming has now been conducted for nearly twenty years throughout the country.

Available data on the impact of the deworming program is limited since no STH-specific community impact assessments have been conducted. Monitoring data are restricted to schistosomiasis co-endemic areas, leaving many districts with no information to guide STH control programming. As a result, the national control program has not changed its treatment strategy since its inception almost twenty years ago. With much of the associated costs of PC programs related to distribution [11], collecting and utilizing survey data to better understand the current epidemiology will likely reduce program delivery costs as fewer rounds of PC are conducted. In this survey, we sought to generate evidence to inform programmatic decision-making related to STH prevention and treatment in groups most at risk for STH-associated morbidity.

### Objectives

The primary objective of these surveys was to estimate (i) the prevalence of any intensity infection and (ii) the prevalence of moderate-to-heavy intensity infection due to *A*. *lumbricoides*, *T*. *trichiura*, and hookworm species (*N*. *americanus* and *A*. *duodenale*) among preschool-aged children (PSAC, 1–4 years old), school-aged children (SAC, 5–14 years old), and women of reproductive age (WRA, 15–49 years old). Our secondary objective was to characterize the populations regarding recent deworming; closed shoe use; and water, sanitation, and hygiene (WaSH) practices.

## Methods

### Ethics statement

The methods used in this survey were reviewed and approved by the Vector Control Division Research Ethics Committee (VCDREC153/4) and the Uganda National Council of Science and Technology (HS1937ES). Permission was received from each of the district and village authorities. Each participant underwent a verbal informed consent or assent process before participating in the survey. Verbal or written consent was obtained from the parent or guardian for child participants.

### Population and setting

**Fig 1:**
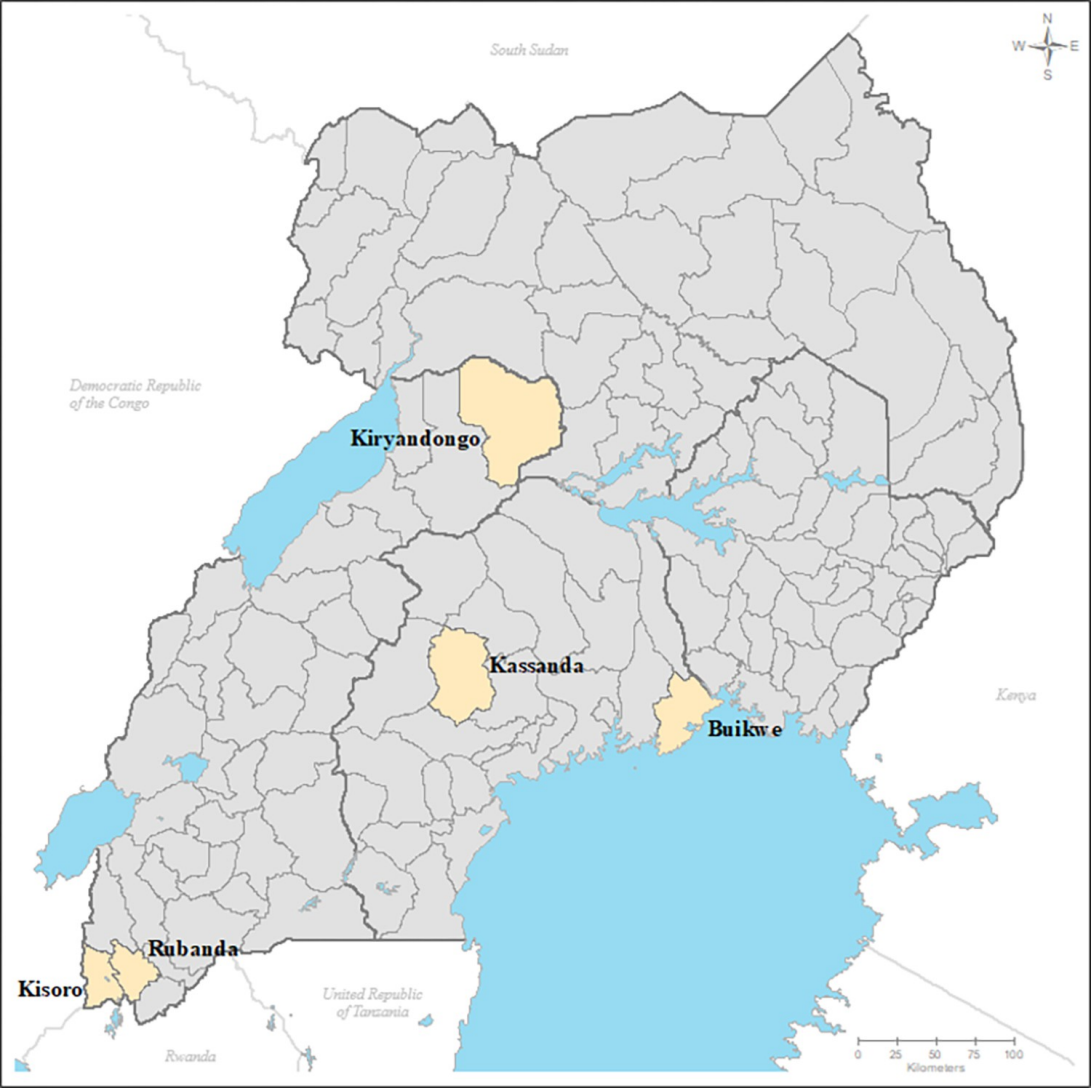
Districts surveyed to assess soil-transmitted helminth infection prevalence during March and May of 2022. *The map was prepared using ArcGIS software using basemap* *https*:*//data*.*humdata*.*org/dataset/cod-ab-uga*.

Surveys were conducted during March (Buikwe) and May (other districts) of 2022, at least six months after the most recent mass PC distribution and directly before the upcoming PC distribution. The target populations of the surveys were PSAC, SAC, and WRA living in the five selected districts. Districts were selected from three ecological areas: Highlands (Rubanda and Kisoro districts), central/Lowland (Kassanda and Kiryandongo districts), and lake shore (Buikwe district). Fig 1 shows the location of the surveyed districts. Note that since baseline surveys were conducted in the 1990s and 2000s, Uganda has sub-divided its 64 districts into 146 smaller administrative units and ten urban special district (cities) and the capital city of Kampala.

All selected districts had conducted twice-a-year deworming programs through child health days with reported coverage of >75% for greater than 10 rounds before the survey. Pregnant women receive deworming tablets through routine health checkups. In schistosomiasis endemic sub-counties in the Buikwe, Kassanda, and Kiryandongo districts, populations may have received additional deworming treatment during schistosomiasis PC. From 1992 to 2018, populations received ivermectin for onchocerciasis (river blindness) in some endemic areas of the Rubanda and Kisoro districts.

### Survey methodology

The survey followed the Integrated Community-based Survey for Program Monitoring (ICSPM) methodology. A manual describing the full details of the ICSPM methodology can be found on the *Children Without Worms* website (https://childrenwithoutworms.org/icspm-reference-manual/). The key methodology is presented below.

### Design

ICSPM is a cross-sectional household survey that utilizes a cluster-sample approach to randomly select communities and households in the survey area to estimate district-level prevalence. In a surveyed district, PSAC, SAC, and WRA have an equal probability of selection within their respective risk group.

### Sampling

A sampling frame was constructed within each district using results from the latest population and housing census. The sampling frame consisted of all clusters in a district and their projected size for the current year in households. A “cluster” was typically equivalent to a village for survey purposes. Villages with less than 100 households were combined with one or more neighboring villages in the same district for sampling purposes to create a single cluster. Each cluster was then divided into segments by dividing the projected number of households by the target segment size (100 households) and rounding to the nearest integer. Thirty clusters were selected with probability proportional to their size in segments.

### Sample size

Per ICSPM methodology, we targeted a sample size of 332 individuals from each risk group in each district to achieve an adequate sample size for STH prevalence threshold testing to <10%. Anticipating that ~40% of enrollees would not submit stool samples, we sought to enroll 553 individuals per risk group.

### Survey conduct

Survey teams consisted of a trained enumerator (member of the Ugandan Ministry of Health), an assistant (member of the district health team), and a volunteer member of the village health team. On each survey day, six field teams conducted surveys in six different clusters (thus the 30 clusters in each district were completed over five days). Village health team volunteers assisted survey teams in community sensitization, mapping, segmenting, and navigating selected villages for enumeration. Enumerators were given lists with each selected household number and the risk group(s) to be enrolled from those households, according to the sampling quotas for each of the groups. A household was defined as a group of people who eat from the same kitchen/cooking pot and a household member was considered a person who lived in that household for more than six months. The survey team enrolled all selected members of the household (which could include multiple members of each risk group).

ICSPM surveys were conducted over two days in each selected cluster. On the first day, field teams visited selected households. After obtaining consent from the head of the household (typically women), the enumerator marked the geolocation of the household and administered a household interview. Consent for children was obtained from the parent/guardian (typically the head of household). The household interview included questions regarding WaSH. Handwashing stations were directly observed by the enumerator while other WaSH factors were self-reported by the head of household. Once individual eligibility was determined, the enumerator conducted an individual interview for each eligible household member by interviewing the head of the household. Any eligible adult household member home at the time of the survey was interviewed directly. The individual interview focused on factors such as demographics, the use of shoes, and the history of deworming.

The survey team distributed stool collection kits with detailed collection instructions to all eligible household members. The kit contained one stool container, old newspaper, and toilet paper for collection. When multiple containers were left at a household, either participant initials for each enrollee (literate head of household) or a series of dots indicating the youngest to oldest enrollee (illiterate head of household) were written on each container. Each stool container was labeled with the QR code corresponding to the individual’s unique assigned code as recorded during the individual interview.

On day two, the village health team volunteer returned to the households to collect all available filled stool containers and provided them to survey staff who returned them immediately to the laboratory (further described below).

### Outcome assessments

The primary outcomes evaluated were the presence of STH infection of any intensity and the presence of moderate-to-heavy intensity infection, measured among PSAC, SAC, and WRA.

### Laboratory assessment

Stool sample containers were put into cooler boxes with two ice packs at the time of collection. For each container, the village health team volunteer recorded the collection time and the reported deposit time. The samples were immediately transported to one of two mobile laboratories for examination. Each of the two laboratories received stool samples from three clusters surveyed the previous day. Laboratory locations were chosen to be near the three assigned clusters.

Upon delivery of stool samples to the laboratory, trained technicians used a smartphone-based ODK form (https://getodk.org/) to scan the QR code on each sample, assesses its quality, and record both the deposit and collection times. Empty or missing containers were also recorded. Samples were processed immediately upon receipt, with samples awaiting processing kept in cooler boxes. All returned samples were included in the laboratory analysis. In rare instances, containers may have leaked, and samples were excluded due to insufficient quantity of stool. Double-slide Kato-Katz microscopy was used to quantify the number of eggs in the stool sample. Slides were read within 60 minutes of preparation per WHO guidelines [12]. On a paper-based form, the microscopists recorded the slide (A or B), the unique identifier of the stool sample, and the egg count per slide of hookworm, *A*. *lumbricoides*, and *T*. *trichiura*. Laboratory information collected on the paper-based form was later entered into an ODK form.

### Classification of infection intensity

The number of eggs per gram of stool was calculated by multiplying the average of the eggs per slide of the two slides by 24 (standard calculation based on 41.7 mg of stool per Kato-Katz smear preparation). In the few instances where only one slide reading was available, the single slide reading was used and multiplied by 24. The eggs per gram estimate was used to classify the samples by infection intensity per WHO guidelines [10].

### Other outcomes assessed

Secondary outcomes included individual and household-level characteristics. Individuals were asked about the type of shoes normally worn, if they had ever swallowed deworming medication, and, if so, how recently and the location of receipt of the pill. Household WaSH indicators were collected and measured, specifically, the proportion of households with an improved source of drinking water, an improved source of non-drinking water, a handwashing station with soap, an improved sanitation facility, and a sanitary method of disposing of the stool of children <5 years of age. Classification of improved status was defined per WHO and UNICEF Joint Monitoring Programme for Water Supply, Sanitation, and Hygiene (https://washdata.org/monitoring/sanitation, https://washdata.org/monitoring/drinking-water).

### Data collection and management

Four electronic ODK survey forms were used in the survey: the household questionnaire, the individual questionnaire, the laboratory specimen intake, and the laboratory specimen results. Data were stored locally on smartphones and uploaded to a cloud-based server when a connection became available. The four forms were linked using household identification numbers and the individual’s unique code. The data manager conducted daily checks for data quality, consistency, and cleaning of submitted data.

### Statistical analyses and software

Within each district and for each risk group, we calculated four estimates of infection prevalence of any intensity: hookworm, *A*. *lumbricoides*, *T*. *trichiura*, and infection caused by any of these species. Similarly, we calculated four estimates of moderate-to-heavy intensity infection prevalence: hookworm, *A*. *lumbricoides*, *T*. *trichiura*, and moderate-to-heavy intensity infection caused by any of these species. For each prevalence estimate, we calculated the design-corrected upper, one-sided 95% confidence limit. Selection weights were not needed given the equal probability of selection within each risk group.

Data were collected using ODK Collect and were cleaned, managed, and analyzed using Stata (College Station, Texas), SAS version 9.4 (Cary, North Carolina), and Microsoft Excel.

## Results

### Survey population

Table 1 shows the number of clusters, households, and individuals enrolled and submitting stool samples by district and risk group. On average, ~ 600 households were enrolled in each district, ranging from a low of 517 in Kisoro to a high of 722 in Buikwe. The proportion of households where at least one member submitted a stool sample was relatively high and ranged from ~ 75 to 90%.

**Table 1:** Number of clusters, households, and individuals surveyed by district and risk group.

District	Clusters*		Households		Individuals			
	**Sur-veyed**	**House-holds per cluster**	**En-rolled**	**Enrolled households where ≥ 1 member submitted a stool sample**	**Risk group**	**En-rolled**	**Among those enrolled, number of individuals submitting a stool sample**	**Among those submitting a stool sample, number female**
	**n**	**mean (range)**	**n**	**n (%)**		**n**	**n (%)**	**n (%)**
**Buikwe**	30	24.1 (16–32)	722	536 (74.2)	**PSAC**	1,050	613 (58.4)	299 (48.8)
					**SAC**	998	629 (63.0)	329 (52.3)
					**WRA**	439	260 (59.2)	260 (100.0)
**Kassanda**	30	19.1 (14–23)	574	474 (82.6)	**PSAC**	1,053	709 (67.3)	346 (48.8)
					**SAC**	1,213	838 (69.1)	436 (52.0)
					**WRA**	289	192 (66.4)	192 (100.0)
**Kiryan-dongo**	30	22.2 (13–38)	665	592 (89.0)	**PSAC**	1,081	821 (75.9)	430 (52.4)
					**SAC**	1,149	916 (79.7)	461 (50.3)
					**WRA**	329	254 (77.2)	254 (100.0)
**Kisoro**	30	17.2 (12–23)	517	426 (82.4)	**PSAC**	970	630 (64.9)	329 (52.2)
					**SAC**	1,234	820 (66.5)	460 (56.1)
					**WRA**	377	260 (69.0)	260 (100.0)
**Rubanda**	30	18.4 (10–24)	551	445 (80.8)	**PSAC**	972	579 (59.6)	296 (51.1)
					**SAC**	1,082	681 (62.9)	361 (53.0)
					**WRA**	434	260 (59.9)	260 (100.0)

Abbreviations: n–Count; PSAC–preschool-aged children (1–4 years old); SAC–school-aged children (5–14 years old); WRA–women of reproductive age (15–49 years old)

*Clusters are typically a single village, however in some cases villages are combined if the projected village size in households is less than the target segment size

Across districts, the number of PSAC submitting stool samples ranged from 579 to 821, SAC ranged from 629 to 916, and WRA ranged from 192 to 260. Response (providing stool sample) averaged ~ 65–68% by risk group, although the response was notably higher in Kiryandongo (~ 76–80%). Among children ages 1–14 years old who submitted stool samples, females tended to submit slightly more frequently than males (51.8% female overall).

### Prevalence estimates

**Fig 2:**
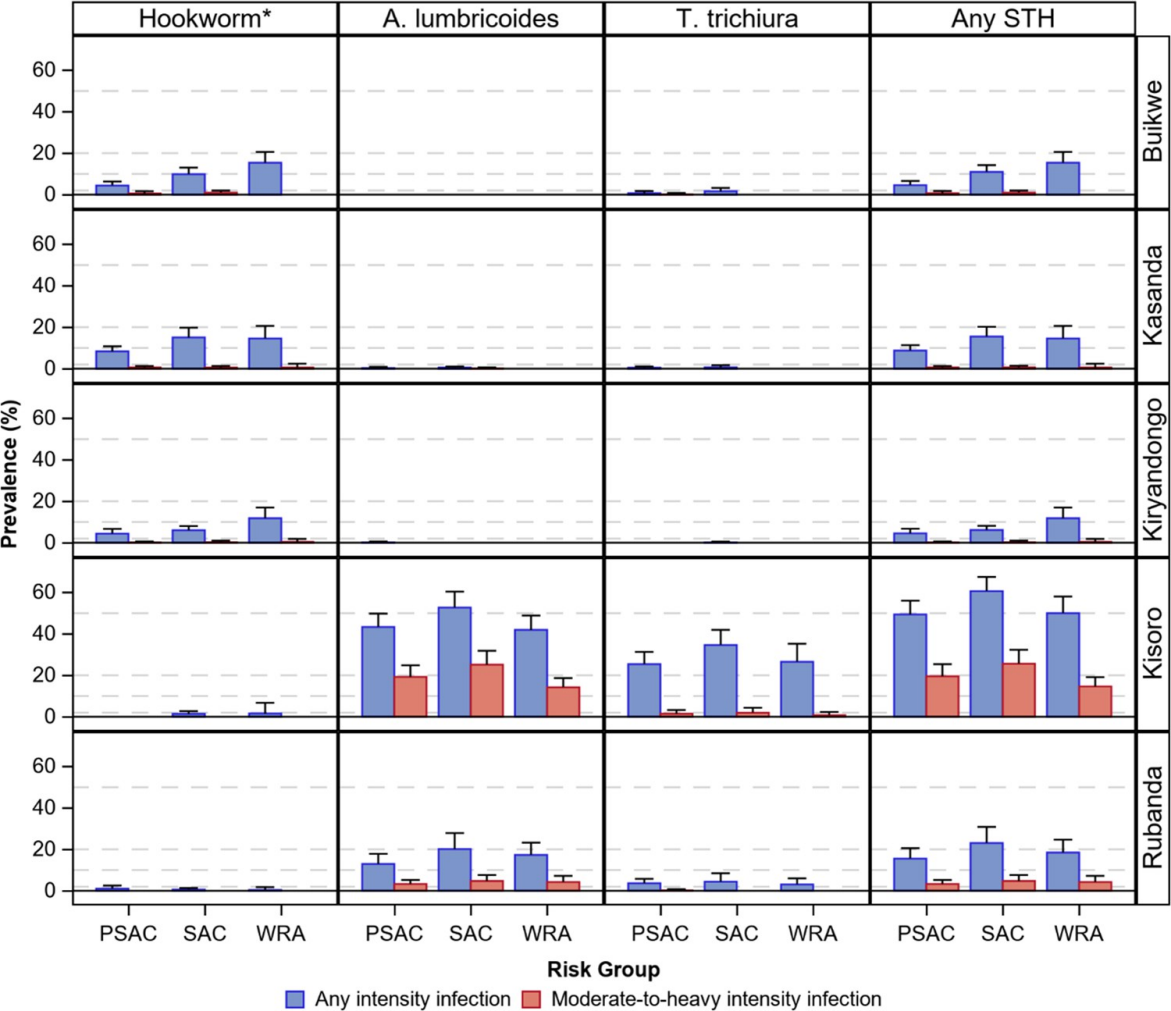
Estimated soil-transmitted helminth infection prevalence by district, risk group, species, and intensity of infection. Abbreviations: STH–Soil-transmitted helminth; PSAC–preschool-aged children (1–4 years old); SAC–school-aged children (5–14 years old); WRA–women of reproductive age (15–49 years old). Error bars represent the upper 95% one-sided confidence limit. Horizontal reference bars (dashed gray lines) represent thresholds for the World Health Organization-recommended preventative chemotherapy distribution frequency. *N. americanus and A. duodenale.

Prevalence estimates by district, species, risk group, and infection intensity are shown in Fig 2 and S1 Table.

### Infections of any intensity

In three districts (Buikwe, Kassanda, Kiryandongo), the prevalence of any intensity STH infection was low to moderate (4.5–15.5%) among all risk groups with infections driven almost exclusively by hookworm. Given this predominant species, the age distribution followed the expected pattern with WRA tending to have the highest prevalence and PSAC the lowest.

In the remaining two districts (Kisoro and Rubanda), we observed a moderate to very high prevalence of any STH. In these Highland districts, infection prevalence ranged from 15.5 to 23.1% by risk group in Rubanda and from 49.4 to 60.6% in Kisoro. Unlike Buikwe, Kassanda, and Kiryandongo, infections in Rubanda and Kisoro were driven primarily by *A*. *lumbricoides* and *T*. *trichiura*. SAC had the highest prevalence of any STH infection, with PSAC and WRA having lower but similar magnitudes.

### Moderate-to-heavy intensity infections

Moderate-to-heavy intensity infection prevalence was ≤1% among all risk groups in Buikwe, Kassanda, and Kiryandongo. Corresponding to the elevated prevalence of any intensity STH infection seen in the Highland districts, moderate-to-heavy intensity infection prevalence was also elevated in Rubanda (~ 3–5%) and Kisoro (~ 15–26%).

### Coinfections

Coinfections were uncommon in Buikwe, Kassanda, Kiryandongo, and Rubanda (Table 2). In Kisoro, *A*. *lumbricoides/T*. *trichiura* coinfection prevalence was high, ranging from 18.1 to 26.1% by risk group. Triple infections were rarely observed.

**Table 2:** Estimated soil-transmitted helminth coinfection prevalence by district and risk group.

District	Risk group	Sub-mitting stool samples	Uninfected		Mono-infection			Co-infection		Triple infection
				Hookworm*	*A*. *lumbri-coides*	*T*. *trichiura*	Hookworm and *A*. *lumbri-coides*	Hookworm and *T*. *trichiura*	*A*. *lumbri-coides* and *T*. *trichiura*	Hookworm, *A*. *lumbri-coides*, and *T*. *trichiura*
		n	n (prevalence)	n (prevalence)	n (prevalence)	n (prevalence)	n (prevalence)	n (prevalence)	n (prevalence)	n (prevalence)
**Buikwe**	**PSAC**	613	585 (95.4)	24 (3.9)	0 (0.0)	1 (0.2)	0 (0.0)	3 (0.5)	0 (0.0)	0 (0.0)
	**SAC**	629	560 (89.0)	59 (9.4)	0 (0.0)	7 (1.1)	0 (0.0)	3 (0.5)	0 (0.0)	0 (0.0)
	**WRA**	260	220 (84.6)	40 (15.4)	0 (0.0)	0 (0.0)	0 (0.0)	0 (0.0)	0 (0.0)	0 (0.0)
**Kassanda**	**PSAC**	709	647 (91.3)	58 (8.2)	1 (0.1)	2 (0.3)	0 (0.0)	0 (0.0)	0 (0.0)	1 (0.1)
	**SAC**	838	708 (84.5)	121 (14.4)	2 (0.2)	2 (0.2)	2 (0.2)	3 (0.4)	0 (0.0)	0 (0.0)
	**WRA**	192	164 (85.4)	28 (14.6)	0 (0.0)	0 (0.0)	0 (0.0)	0 (0.0)	0 (0.0)	0 (0.0)
**Kiryan-dongo**	**PSAC**	821	784 (95.5)	36 (4.4)	1 (0.1)	0 (0.0)	0 (0.0)	0 (0.0)	0 (0.0)	0 (0.0)
	**SAC**	916	860 (93.9)	55 (6.0)	0 (0.0)	1 (0.1)	0 (0.0)	0 (0.0)	0 (0.0)	0 (0.0)
	**WRA**	254	224 (88.2)	30 (11.8)	0 (0.0)	0 (0.0)	0 (0.0)	0 (0.0)	0 (0.0)	0 (0.0)
**Kisoro**	**PSAC**	630	319 (50.6)	0 (0.0)	151 (24.0)	38 (6.0)	0 (0.0)	0 (0.0)	122 (19.4)	0 (0.0)
	**SAC**	820	323 (39.4)	0 (0.0)	209 (25.5)	63 (7.7)	4 (0.5)	2 (0.2)	214 (26.1)	5 (0.6)
	**WRA**	260	130 (50.0)	1 (0.4)	59 (22.7)	20 (7.7)	1 (0.4)	0 (0.0)	47 (18.1)	2 (0.8)
**Rubanda**	**PSAC**	579	489 (84.5)	3 (0.5)	64 (11.1)	11 (1.9)	2 (0.3)	1 (0.2)	9 (1.6)	0 (0.0)
	**SAC**	681	524 (76.9)	1 (0.1)	125 (18.4)	18 (2.6)	1 (0.1)	1 (0.1)	10 (1.5)	1 (0.1)
	**WRA**	260	212 (81.5)	0 (0.0)	40 (15.4)	2 (0.8)	0 (0.0)	1 (0.4)	5 (1.9)	0 (0.0)

Abbreviations: n–Count; PSAC–preschool-aged children (1–4 years old); SAC–school-aged children (5–14 years old); WRA–women of reproductive age (15–49 years old)

*N. americanus and A. duodenale

### Other infections identified

*Hymenolepis nana*, *Taenia spp*., and *Enterobius vermicularis* were identified infrequently, with the prevalence of infection <1% for each species among all risk groups, except for *H*. *nana* among SAC, where the prevalence reached 1.3%.

### Population characteristics

#### Recent deworming

**Fig 3:**
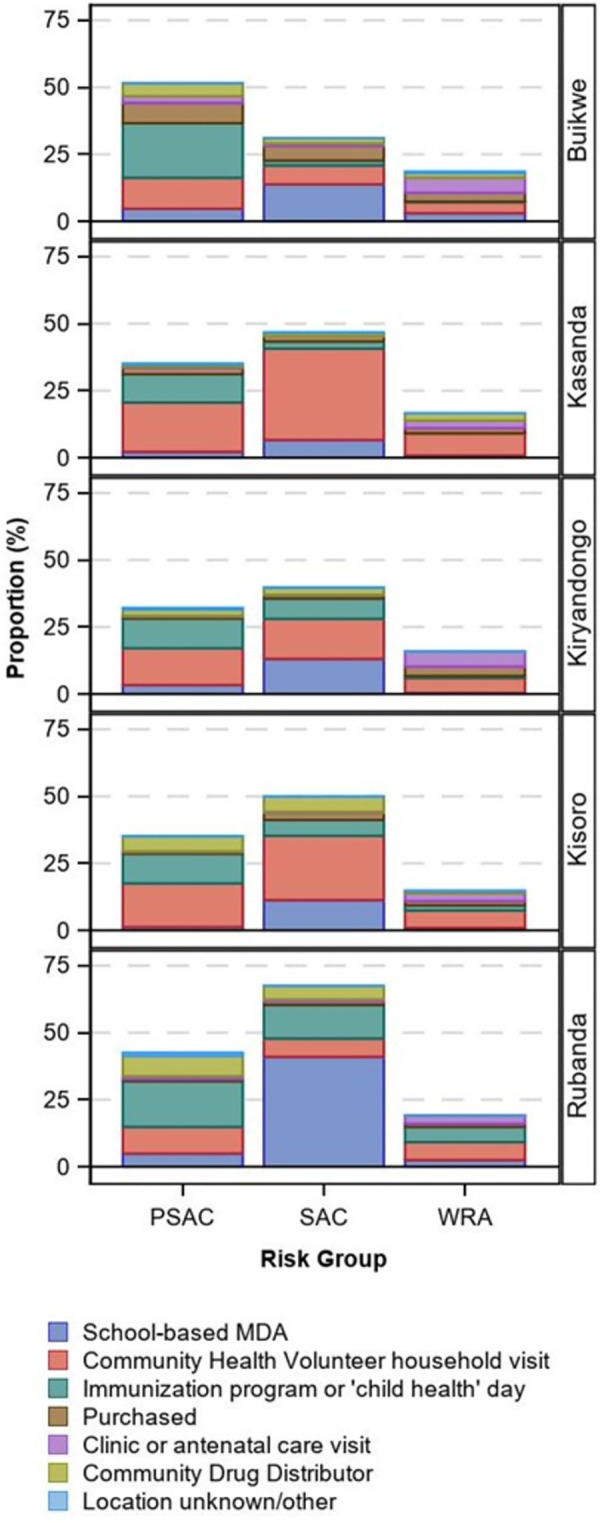
Proportion of individuals reporting swallowing deworming medication within the last six months by location of receipt, district, and risk group.

The history of "recent” (within the last six months) deworming is shown in Table 3 and Fig 3. Among children in Buikwe, PSAC most reported receiving recent deworming (51.5%), followed by SAC (31.0%). However, in all other districts, SAC most commonly reported recent swallowing of deworming medications, ranging from a low of 39.8% in Kiryandongo to a high of 67.5% in Rubanda. PSAC were treated slightly less frequently than SAC and fewer than 20% of WRA reported recent deworming in all districts.

**Table 3:** The proportion of individuals reporting swallowing deworming medication within the last six months by district, risk group, and location of receipt.

District	Risk group	En-rolled	Yes							No	Un-known
			School-based MDA	Commun-ity Health Volunteer household visit	Immuniza-tion program or ’Child Health Day’	Purchased	Clinic or antenatal care visit	Commun-ity Drug Distributor	Other or unspecified location		
		n	n (%)	n (%)	n (%)	n (%)	n (%)	n (%)	n (%)	n (%)	n
**Buikwe**	**PSAC**	1,050	45 (4.7)	111 (11.6)	195 (20.3)	73 (7.6)	24 (2.5)	46 (4.8)	0 (0.0)	465 (48.5)	91
	**SAC**	998	126 (13.8)	64 (7.0)	17 (1.9)	50 (5.5)	8 (0.9)	18 (2.0)	0 (0.0)	629 (69.0)	86
	**WRA**	439	12 (3.1)	16 (4.1)	1 (0.3)	13 (3.3)	22 (5.6)	7 (1.8)	2 (0.5)	320 (81.4)	46
**Kassanda**	**PSAC**	1,053	20 (2.2)	169 (18.4)	97 (10.6)	22 (2.4)	3 (0.3)	9 (1.0)	3 (0.3)	596 (64.9)	134
	**SAC**	1,213	72 (6.6)	371 (34.1)	29 (2.7)	23 (2.1)	0 (0.0)	13 (1.2)	2 (0.2)	579 (53.2)	124
	**WRA**	289	2 (0.8)	21 (8.3)	0 (0.0)	5 (2.0)	7 (2.8)	7 (2.8)	0 (0.0)	211 (83.4)	36
**Kiryan-dongo**	**PSAC**	1,081	31 (3.3)	128 (13.8)	103 (11.1)	7 (0.8)	2 (0.2)	22 (2.4)	6 (0.6)	631 (67.9)	151
	**SAC**	1,149	139 (13.1)	159 (15.0)	80 (7.5)	14 (1.3)	1 (0.1)	28 (2.6)	2 (0.2)	639 (60.2)	87
	**WRA**	329	1 (0.4)	14 (5.5)	2 (0.8)	9 (3.6)	14 (5.5)	0 (0.0)	0 (0.0)	213 (84.2)	76
**Kisoro**	**PSAC**	970	10 (1.2)	131 (16.3)	89 (11.1)	5 (0.6)	2 (0.3)	45 (5.6)	1 (0.1)	521 (64.8)	166
	**SAC**	1,234	124 (11.3)	264 (24.0)	65 (5.9)	29 (2.6)	4 (0.4)	63 (5.7)	1 (0.1)	550 (50.0)	134
	**WRA**	377	3 (1.0)	20 (6.4)	6 (1.9)	5 (1.6)	9 (2.9)	3 (1.0)	0 (0.0)	265 (85.2)	66
**Rubanda**	**PSAC**	972	40 (4.9)	81 (9.9)	139 (17.1)	7 (0.9)	8 (1.0)	62 (7.6)	10 (1.2)	468 (57.4)	157
	**SAC**	1,082	413 (41.0)	69 (6.9)	126 (12.5)	13 (1.3)	8 (0.8)	51 (5.1)	0 (0.0)	328 (32.5)	74
	**WRA**	434	10 (2.5)	27 (6.7)	23 (5.7)	5 (1.2)	12 (3.0)	0 (0.0)	0 (0.0)	327 (80.9)	30

Abbreviations: n–Count; PSAC–preschool-aged children (1–4 years old); SAC–school-aged children (5–14 years old); WRA–women of reproductive age (15–49 years old); MDA–mass drug administration

Percentages exclude unknown responses

Regarding the location where those who were recently treated received the deworming medication, Community Health Volunteer household visits were most reported among all risk groups in Kassanda, Kiryandongo, and Kisoro (although clinic/antenatal care was equally high in Kiryandongo among WRA). Among children in Buikwe and Rubanda, receipt at immunization program/ ’child health’ day was most common among PSAC, and receipt via school-based mass PC distribution among SAC. WRA were treated primarily through clinics/antenatal care in Buikwe and at Community Health Volunteer household visits in Rubanda.

### Shoe use

**Fig 4:**
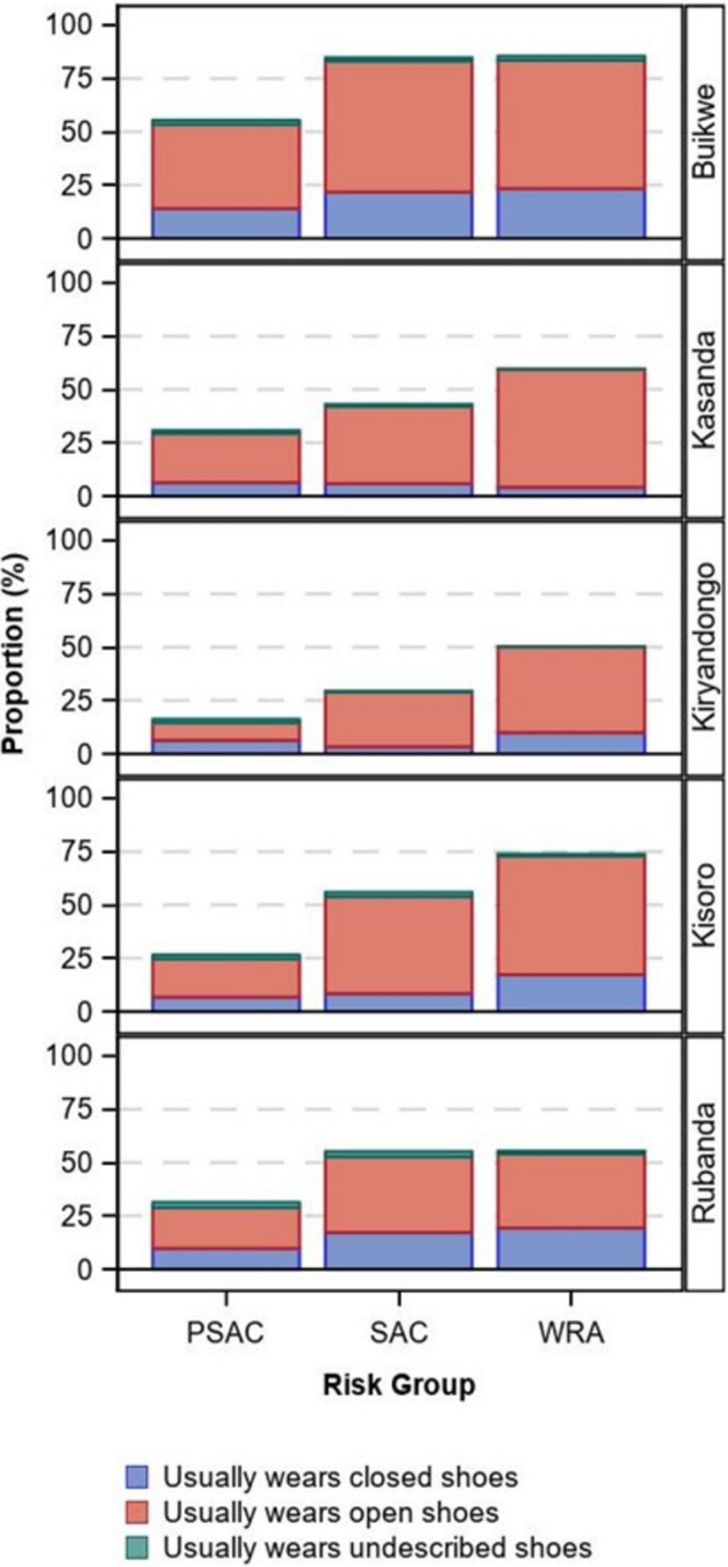
Proportion of individuals reporting shoe use by type, district, and risk group.

In all districts, the usual use of shoes was lowest among PSAC (district range 16.3–55.4%), followed by SAC (29.4–84.7%), and then WRA (50.2–85.4%, Table 4 and Fig 4). Buikwe tended to have higher overall shoe usage, while the remaining districts were similar. When worn, open shoes were most used.

**Table 4:** Proportion of individuals reporting shoe use by district, risk group, and shoe type.

District	Risk group	Enrolled	Does not usually wear shoes	Usually wears closed shoes	Usually wears open-toed/ sided shoes	Usually wears shoes, type not described	Unknown
		n	n (%)	n (%)	n (%)	n (%)	n
**Buikwe**	**PSAC**	1,050	416 (44.6)	131 (14.0)	368 (39.4)	18 (1.9)	117
	**SAC**	998	141 (15.3)	200 (21.7)	565 (61.4)	14 (1.5)	78
	**WRA**	439	58 (14.6)	93 (23.4)	239 (60.1)	8 (2.0)	41
**Kassanda**	**PSAC**	1,053	631 (69.2)	58 (6.4)	210 (23.0)	13 (1.4)	141
	**SAC**	1,213	625 (57.0)	65 (5.9)	397 (36.2)	10 (0.9)	116
	**WRA**	289	107 (40.5)	11 (4.2)	146 (55.3)	0 (0.0)	25
**Kiryandongo**	**PSAC**	1,081	739 (83.7)	56 (6.3)	73 (8.3)	15 (1.7)	198
	**SAC**	1,149	738 (70.6)	35 (3.3)	268 (25.6)	4 (0.4)	104
	**WRA**	329	145 (49.8)	29 (10.0)	117 (40.2)	0 (0.0)	38
**Kisoro**	**PSAC**	970	616 (73.4)	57 (6.8)	150 (17.9)	16 (1.9)	131
	**SAC**	1,234	497 (44.1)	95 (8.4)	512 (45.4)	23 (2.0)	107
	**WRA**	377	91 (26.2)	60 (17.3)	193 (55.6)	3 (0.9)	30
**Rubanda**	**PSAC**	972	583 (68.6)	83 (9.8)	163 (19.2)	21 (2.5)	122
	**SAC**	1,082	453 (44.8)	174 (17.2)	359 (35.5)	25 (2.5)	71
	**WRA**	434	184 (44.6)	80 (19.4)	143 (34.6)	6 (1.5)	21

Abbreviations: n–Count; PSAC–preschool-aged children (1–4 years old); SAC–school-aged children (5–14 years old); WRA–women of reproductive age (15–49 years old)

Percentages exclude unknown responses

### Water, sanitation, and hygiene

Household-level WaSH measures are shown in Table 5. The proportion of households using improved drinking water sources ranged from 65.2 to 94.6%, being lowest in Kassanda and highest in Buikwe. A similar pattern was observed for households using an improved water source for other purposes. Less than 10% of households had a handwashing station with soap in all districts except Buikwe (17.7%).

**Table 5:** Proportion of households reporting use of select water, sanitation, and hygiene practices by district.

Water/hygiene							
District	House-holds	Improved* source of drinking water		Improved source of non-drinking water		Handwashing station with soap observed	
		Yes	Unknown	Yes	Unknown	Yes	Unknown/ refused
	n	n (%)	n	n (%)	n	n (%)	n
**Buikwe**	722	683 (94.6)	0	687 (95.4)	2	127 (17.7)	3
**Kassanda**	574	371 (65.2)	5	332 (57.9)	1	22 (4.0)	28
**Kiryandongo**	665	547 (82.6)	3	510 (77.4)	6	23 (3.8)	53
**Kisoro**	517	404 (78.4)	2	391 (76.1)	3	44 (8.7)	12
**Rubanda**	551	441 (80.3)	2	443 (80.7)	2	30 (5.5)	2
**Sanitation**							
**District**	**House-holds**	**Improved status of sanitation facility normally used**				**Sanitary disposal of child’s stool** ^**§**^	
		**Improved** ^**†**^	**Unimproved** ^**‡**^	**No facilities**	**Unknown**	**Yes**	**Unknown/ no children**
	**n**	**n (%)**	**n (%)**	**n (%)**	**n**	**n (%)**	**n**
**Buikwe**	722	447 (62.0)	272 (37.7)	2 (0.3)	1	621 (98.3)	90
**Kassanda**	574	204 (36.0)	349 (61.7)	13 (2.3)	8	360 (77.3)	108
**Kiryandongo**	665	180 (27.2)	473 (71.5)	9 (1.4)	3	480 (79.9)	64
**Kisoro**	517	152 (29.6)	353 (68.8)	8 (1.6)	4	357 (83.6)	90
**Rubanda**	551	136 (24.7)	410 (74.5)	4 (0.7)	1	433 (91.5)	78

Abbreviations: n–Count

Percentages exclude unknown responses

*Improved water sources include piped water, boreholes or tube wells, protected dug wells, protected springs, rainwater, and packaged or delivered water

^†^Improved sanitation facilities include flush/pour flush toilets connected to piped sewer systems, septic tanks or pit latrines; pit latrines with slabs (including ventilated pit latrines), and composting toilets

^‡^Unimproved sanitation facilities include pit latrines without slabs, hanging latrines, and bucket latrines

^§^Children include those ages 0–4 years; sanitary disposal includes placing in the toilet, compost, or trash pit (versus burying or throwing outside)

Household-level improved sanitation coverage was low and <40% in all districts except Buikwe (62.0%). However, all districts had household coverages of >97% for some type (improved or unimproved) of sanitation facility. Over 75% of all households in each district disposed of the stool of children ages 0–4 years in the toilet, compost, or trash pit.

## Discussion

As compared with the previous school-based studies conducted in Uganda [7,8], results from our community-based surveys suggest that the prevalence of STH infection has markedly decreased in the Buikwe, Kassanda, and Kiryandongo districts among all assessed risk groups (PSAC, SAC, and WRA). Despite decades of deworming, however, the prevalence remained moderate to high in Rubanda and very high in Kisoro; further investigation is needed to understand why.

### Evidence to guide STH control programming

Moving away from PC coverage-based targets, WHO has shifted its focus for STH control programs to eliminating morbidity due to STH infections as a public health problem, defined as a moderate-to-heavy intensity infection prevalence due to any STH of <2% among children [2,13]. Our results indicate that this has been achieved in Buikwe, Kassanda, and Kiryandongo. However, per WHO PC frequency recommendations following years of deworming [10,11], PC is still warranted once-a-year or once every two years in these areas. If adopted, this shift would mark a substantial reduction in PC frequency from the current twice-a-year distribution in these areas, potentially freeing donated drugs and resources to be allocated to those who remain at the highest risk of morbidity.

On the contrary, in the Highland districts of Kisoro and Rubanda, our results indicated that elimination as a public health problem has yet to be achieved. The district of Rubanda was close to achieving this target however, with moderate-to-heavy intensity infection prevalence less than 2 and 3 percentage points above the 2% threshold among PSAC and SAC, respectively. Continued twice-a-year or reduced once-a-year PC is indicated in Rubanda.

In high prevalence Kisoro, we found that approximately one out of every four SAC, one out of every five PSAC, and one out of every six WRA had a moderate-to-heavy intensity infection due to any STH parasite, indicating that a significant proportion of the population remains at risk for STH-associated morbidity. Per WHO recommendations, PC should be distributed two to three times per year in this district, however urgent attention is also needed to determine why the deworming program has not resulted in similar gains in control as seen in other surveyed districts. Our results did not suggest that fewer were getting dewormed, as the reported frequency of recent deworming (within six months) was similar to other surveyed districts. We hypothesize that in the mountainous regions of Kisoro, where Community Health household visits are a predominant method of reaching populations, hard-to-reach populations may be difficult to access and thus may not receive regular deworming. While high treatment coverage has been reported over the years, an independent evaluation has yet to be conducted to confirm the reported coverages and our results suggest a need to validate the reported coverage in the district. In addition, unlike the Lowland districts where schistosomiasis control programs operate using ‘grassroots’ efforts, Kisoro and Rubanda districts lack these additional, effective delivery channels.

The dominant parasites identified in Kisoro and Rubanda may also help explain the findings. Like the distribution observed in previous work [7,8], survey findings showed that hookworm infections were more common in the Lowlands, while *A*. *lumbricoides and T*. *trichiura* infections remained the most prevalent in the Highlands (Kisoro and Rubanda). Importantly, *T*. *trichiura* infections reached an estimated prevalence of ~ 35% among SAC in Kisoro, with PSAC and WRA prevalence close behind. Given the limited efficacy of single-dose albendazole and mebendazole against *T*. *trichiura* [14], further consideration of the potential use of combination therapies is warranted to improve egg reduction in this *T*. *trichiura*-dominant area. Finally, anthelminthic drug resistance—not yet conclusively demonstrated in human populations—should be considered if other programmatic insufficiencies are not identified that provide more likely explanations for this finding in Kisoro.

### WaSH use

Along with periodic PC distribution, long-term improvements to WaSH are believed to be vital to both reaching and sustaining gains in STH control even after elimination as a public health problem has been achieved. While the evidence supporting the role of WaSH in STH prevention is mixed [3–6], the use of sanitary facilities that hygienically separate excreta from human contact is (in theory) crucial to not continuously contaminate the environment. At the same time improved water sources are similarly crucial to remove contaminated soil from hands and foods to avoid ingestion of eggs.

In the five surveyed districts, we found that the use of improved sources of drinking water varied by district but was approaching the Sustainable Development Goal Target 6.1 (https://sdgs.un.org/goals/goal6) of universal access in some districts (e.g., Buikwe with a coverage of 94.7%). On the contrary, while open defecation was rare in surveyed districts, the use of improved sanitation facilities remains uncommon, with less than 40% of households reporting use in all districts except Buikwe (>60%). At the current improved latrine coverages, communities may remain at risk of not achieving 2030 targets—and of STH recrudescence once targets are achieved—if WaSH is not strengthened in these districts.

### Survey optimization

As STH control programs move away from universal PC distribution to all at-risk children and towards data-driven distribution to those who remain most at risk of STH-associated morbidity, strengthening monitoring and evaluation systems will be crucial to enhance programming and PC distribution. Survey teams exceeded enrollment targets in these surveys. We believe this was due to two reasons. First, we assumed smaller household sizes in the districts than was encountered. In future surveys, to minimize over-enrollment due to inaccurate sampling assumptions, household size estimates from local and district level officials should be used for survey planning. The second reason we believe we exceeded enrollment targets was due to effective planning and sensitization of the communities. Prior to the surveys, we communicated extensively with district level officials and local communities. We believe that house-to-house mobilization and enrollment with the support of the district health officials and local village health teams, improved the participants’ responsiveness. We also believe that setting up mobile laboratory teams near the surveyed villages gave the volunteer member of the village health team ample time to reach all of the enrolled households and allowed for rapid delivery of the stool samples to the lab. With this approach, 13/15 risk groups surveyed met or exceeded the stool submission expectation of 60%.

### Limitations

This survey has limitations. First, as just mentioned, survey response (returning a stool sample after enrolling in the survey) was ~ 65–70% by risk group. While sub-optimal, this percentage aligns with other similar STH community-based surveys [15]. Infection status among those who are un- or lightly infected (i.e., asymptomatic) can reasonably be assumed not to affect survey participation, however, symptomatic individuals with moderate-to-heavy intensity infections (i.e., potentially symptomatic) may participate at higher proportions, thus potentially overestimating the prevalence of any intensity and moderate-to-heavy intensity infections. Second, we used a single stool sample for microscopic examination using the duplicate Kato Katz method. Kato-Katz is understood to sub-optimally detect light infections, and, together with the known variation in daily egg output [16,17] and the heterogeneity of egg distribution within a single stool sample [18], the prevalence may be underestimated. Finally, hookworm eggs are susceptible to rapid degradation [19], especially in warm, humid climates. While sample integrity received significant attention from staff (e.g., stools were kept in coolers until slide preparation began and laboratory staff read prepared slides within 60 minutes of preparation), eggs may still have degraded. However, given our careful attention to preserving the integrity and given that results corresponded to the expected distribution of hookworm based on previous studies [7,8], it is unlikely this is a great threat to the validity of the hookworm estimates.

### Conclusions

In conclusion, as global STH control programs have evolved and the burden of STH has presumably decreased substantially, a move to evidence-driven programming has become increasingly important to ensure that deworming drugs and other interventions reach those most in need. Using evidence from these surveys, the Ugandan national deworming program may consider changes from the current twice-a-year distribution of preventative chemotherapy in some districts where estimates suggest the prevalence has markedly decreased. A reduction in deworming drug needs in these districts may allow for strengthening targeted treatment for WRA and PSAC nationally. At the same time, further work is needed to enhance the STH control program in Kisoro and Rubanda so that 2030 targets can be reached for all Ugandans at risk for STH-associated morbidity.

## Supporting information

S1 Table Estimated soil-transmitted helminth infection prevalence by district, risk group, species, and intensity of infection. (DOCX)
