# Correction: Prevalence and intensity of soil-transmitted helminth infections in Uganda: Results from population-based prevalence surveys in five districts

**DOI:** 10.1371/journal.pntd.0011804

**Published:** 2023-12-06

**Authors:** The Neglected Tropical Diseases Staff

The funding statement was listed incorrectly. It should read as follows:

“This research was funded by a grant provided to Children Without Worms (Georgia, USA) by Johnson and Johnson (New Jersey, USA) to support surveys conducted by the Vector-borne and Neglected Tropical Disease Division, Ministry of Health, Uganda. The funders had no role in the design of the study design, data collection, data analysis, or interpretation. The findings and conclusion of the study reflect the view of the authors only.”
