# Supplementary material for: Prevalence and intensity of soil-transmitted helminth infections in Uganda: Results from population-based prevalence surveys in five districts
Source: PLoS Negl Trop Dis. 2023 Sep 26;17(9):e0011605. doi: 10.1371/journal.pntd.0011605 (PMC10522024; doi:10.1371/journal.pntd.0011605)
Supplement: S1 Table — (DOCX) [file pntd.0011605.s001.docx]

| **S1 Table. Estimated soil-transmitted helminth infection prevalence by district, risk group, species, and intensity of infection** | | | | | | | |  |
| --- | --- | --- | --- | --- | --- | --- | --- | --- |
| **District** | **Risk group** | **Submitting stool samples** | **Hookworm^*^** | ***A. lumbricoides*** | ***T. trichiura*** | **Any STH** | **PC frequency/ EPHP status^†^** |  |
|  |  | **n** | **prevalence (95% UCL)** | **prevalence (95% UCL)** | **prevalence (95% UCL)** | **prevalence (95% UCL)** |  |  |
| **Any intensity infections** | | | | | | | |  |
| **Buikwe** | **PSAC** | 613 | 4.4 (6.3) | 0.0 (--) | 0.7 (1.7) | 4.6 (6.6) | 1x/2 years |  |
|  | **SAC** | 629 | 9.9 (13.0) | 0.0 (--) | 1.6 (3.2) | 11.0 (14.2) | 1x/year |  |
|  | **WRA** | 260 | 15.4 (20.6) | 0.0 (--) | 0.0 (--) | 15.4 (20.6) | 1x/year |  |
| **Kassanda** | **PSAC** | 709 | 8.3 (10.8) | 0.3 (0.9) | 0.4 (1.1) | 8.7 (11.3) | 1x/2 years |  |
|  | **SAC** | 838 | 15.0 (19.8) | 0.5 (1.1) | 0.6 (1.6) | 15.5 (20.2) | 1x/year |  |
|  | **WRA** | 192 | 14.6 (20.6) | 0.0 (--) | 0.0 (--) | 14.6 (20.6) | 1x/year |  |
| **Kiryandongo** | **PSAC** | 821 | 4.4 (6.7) | 0.1 (0.6) | 0.0 (--) | 4.5 (6.8) | 1x/2 years |  |
|  | **SAC** | 916 | 6.0 (8.0) | 0.0 (--) | 0.1 (0.5) | 6.1 (8.1) | 1x/2 years |  |
|  | **WRA** | 254 | 11.8 (17.0) | 0.0 (--) | 0.0 (--) | 11.8 (17.0) | 1x/year |  |
| **Kisoro** | **PSAC** | 630 | 0.0 (--) | 43.3 (49.8) | 25.4 (31.3) | 49.4 (56.0) | 2x/year |  |
|  | **SAC** | 820 | 1.3 (2.7) | 52.7 (60.4) | 34.6 (41.9) | 60.6 (67.4) | 3x/year |  |
|  | **WRA** | 260 | 1.5 (6.7) | 41.9 (48.8) | 26.5 (35.2) | 50.0 (58.0) | 3x/year |  |
| **Rubanda** | **PSAC** | 579 | 1.0 (2.5) | 13.0 (17.8) | 3.6 (5.7) | 15.5 (20.5) | 1x/year |  |
|  | **SAC** | 681 | 0.6 (1.3) | 20.1 (27.9) | 4.4 (8.5) | 23.1 (30.8) | 2x/year |  |
|  | **WRA** | 260 | 0.4 (1.8) | 17.3 (23.3) | 3.1 (6.0) | 18.5 (24.6) | 1x/year |  |
|  |  |  |  |  |  |  | *(continued)* |  |
| **District** | **Risk group** | **Submitting stool samples** | **Hookworm^*^** | ***A. lumbricoides*** | ***T. trichiura*** | **Any STH** | **PC frequency/ EPHP status^†^** |  |
|  |  | **n** | **prevalence (95% UCL)** | **prevalence (95% UCL)** | **prevalence (95% UCL)** | **prevalence (95% UCL)** |  |  |
| **Moderate-to-heavy intensity infections** | | | | | | | |  |
| **Buikwe** | **PSAC** | 613 | 0.5 (1.6) | 0.0 (--) | 0.2 (0.8) | 0.7 (1.7) | Met EPHP |  |
|  | **SAC** | 629 | 1.0 (2.0) | 0.0 (--) | 0.0 (--) | 1.0 (2.0) | Met EPHP |  |
|  | **WRA** | 260 | 0.0 (--) | 0.0 (--) | 0.0 (--) | 0.0 (--) | N/A |  |
| **Kassanda** | **PSAC** | 709 | 0.6 (1.3) | 0.0 (--) | 0.0 (--) | 0.6 (1.3) | Met EPHP |  |
|  | **SAC** | 838 | 0.5 (1.3) | 0.1 (0.6) | 0.0 (--) | 0.6 (1.4) | Met EPHP |  |
|  | **WRA** | 192 | 0.5 (2.4) | 0.0 (--) | 0.0 (--) | 0.5 (2.4) | N/A |  |
| **Kiryandongo** | **PSAC** | 821 | 0.1 (0.6) | 0.0 (--) | 0.0 (--) | 0.1 (0.6) | Met EPHP |  |
|  | **SAC** | 916 | 0.2 (1.0) | 0.0 (--) | 0.0 (--) | 0.2 (1.0) | Met EPHP |  |
|  | **WRA** | 254 | 0.4 (1.8) | 0.0 (--) | 0.0 (--) | 0.4 (1.8) | N/A |  |
| **Kisoro** | **PSAC** | 630 | 0.0 (--) | 19.2 (24.8) | 1.4 (3.3) | 19.5 (25.4) | Not yet met EPHP |  |
|  | **SAC** | 820 | 0.0 (--) | 25.1 (31.8) | 2.0 (4.4) | 25.6 (32.3) | Not yet met EPHP |  |
|  | **WRA** | 260 | 0.0 (--) | 14.2 (18.7) | 0.8 (2.3) | 14.6 (19.1) | N/A |  |
| **Rubanda** | **PSAC** | 579 | 0.0 (--) | 3.3 (5.3) | 0.2 (0.8) | 3.3 (5.3) | Not yet met EPHP |  |
|  | **SAC** | 681 | 0.0 (--) | 4.7 (7.6) | 0.0 (--) | 4.7 (7.6) | Not yet met EPHP |  |
|  | **WRA** | 260 | 0.0 (--) | 4.2 (7.2) | 0.0 (--) | 4.2 (7.2) | N/A |  |
| *Abbreviations: n–Count; UCL–Upper 95% one-sided confidence limit; STH–Soil-transmitted helminth; PC–Preventative chemotherapy; EPHP–Elimination as a public health problem; PSAC–preschool-aged children (1–4 years old); SAC–school-aged children (5–14 years old); WRA–women of reproductive age (15–49 years old)* | | | | | | | | |
| *‘--' indicates no infections of this type so confidence limit not calculated* | | | | | | | | |
| *^*^N. americanus and A. duodenale* | | | | | | | | |
| *^†^PC frequency recommendations are based on the prevalence of any STH infection of any intensity with thresholds at 2%, 10%, 20%, and 50% (Source: Preventive Chemotherapy to Control Soil-Transmitted Helminth Infections in At-Risk Population Groups (2017)). EPHP is defined as a <2% moderate-to-heavy intensity infection prevalence due to any STH among PSAC and SAC (Source: 2030 Targets for Soil-Transmitted Helminthiases Control Programmes (2020)). EPHP is not applicable to WRA.* | | | | | | | | |
